# Diagnostic dilemma of cystic biliary atresia: A series of two cases and brief review of the diagnostic modalities

**DOI:** 10.1002/jpr3.70137

**Published:** 2025-12-26

**Authors:** Hamza Hassan Khan, Leslie Hirsig Spence, Nagraj Kasi

**Affiliations:** ^1^ Division of Pediatric Gastroenterology, Department of Pediatrics, Shawn Jenkins Children's Hospital Medical University of South Carolina Charleston South Carolina USA; ^2^ Division of Pediatric Gastroenterology, Department of Pediatrics, Arkansas Children's Hospital University of Arkansas for Medical Sciences Little Rock Arkansas USA; ^3^ Division of Pediatric Radiology, Department of Radiology, Shawn Jenkins Children's Hospital Medical University of South Carolina Charleston South Carolina USA

**Keywords:** choledochal cyst, direct hyperbilirubinemia, Kasai hepatoportoenterostomy, pediatric gastroenterology, pediatric radiology

## Abstract

Cystic biliary atresia (CBA) is a rare variant of biliary atresia that closely resembles choledochal cyst (CC), complicating diagnosis and potentially delaying critical surgical intervention. We report two cases of CBA that were difficult to diagnose. Case 1 involved a 32^+2^‐week‐old infant with ABO incompatibility and conjugated hyperbilirubinemia. Ultrasound suggested CC, but magnetic resonance cholangiopancreatography (MRCP) indicated CBA, confirmed by intraoperative cholangiogram. The infant underwent Kasai hepatoportoenterostomy (KHPE) and is now 2 years old with normal liver function. Case 2 involved a 17‐day‐old infant with hyperbilirubinemia and pigmented stools. Ultrasound and MRCP suggested CC or CBA, confirmed as CBA through intraoperative cholangiogram. The infant underwent KHPE but later required liver transplantation due to cirrhosis and is now 2 years old with normal liver function. Early diagnosis and timely surgical intervention are crucial for managing CBA, with KHPE being the treatment of choice if performed within 30–45 days of life.

## Abstract (highlights)

What is Known

- Cystic biliary atresia (CBA) is a rare variant of biliary atresia and can mimic choledochal cyst (CC) on imaging.
- Early diagnosis and timely Kasai hepatoportoenterostomy (KHPE) are critical for preserving native liver function.
- Magnetic resonance cholangiopancreatography (MRCP) and ultrasound are commonly used in the evaluation of neonatal cholestasis.

What is New

- We report two cases where MRCP with Eovist raised suspicion for CBA, later confirmed by intraoperative cholangiogram.
- CBA may present with imaging findings similar to CC, underscoring the need for high clinical suspicion.
- Timely distinction between CBA and CC is vital to guide surgical management.

## 1 INTRODUCTION

Biliary atresia (BA) is a rare, progressive, obstructive, fibrosing cholangiopathy that affects the biliary system in approximately 1 in 10,000–15,000 newborns in the United States.^1^ It is the most common indication for pediatric liver transplantation.^2^ Hence, early diagnosis and prompt early intervention are critical for long‐term prognosis of these patients; Kasai hepatoportoenterostomy (KHPE) is the primary surgical intervention for BA, and outcomes are optimal when performed within 30–45 days of life.^3^ Of the morphologic types of BA, cystic BA (CBA) is a relatively uncommon variant of BA, accounting for 5%–10% of the BA cases and mimicking choledochal cyst (CC) in clinical presentation, leading to diagnostic dilemma.^4^ We report a series of two cases that resembled CC on initial imaging studies but were later found to be CBA on confirmatory studies.

## 2 CASE REPORT

### 2.1 Ethics statement

Informed consent was obtained from the respective guardians.

### 2.2 Case 1

**Figure 1:**
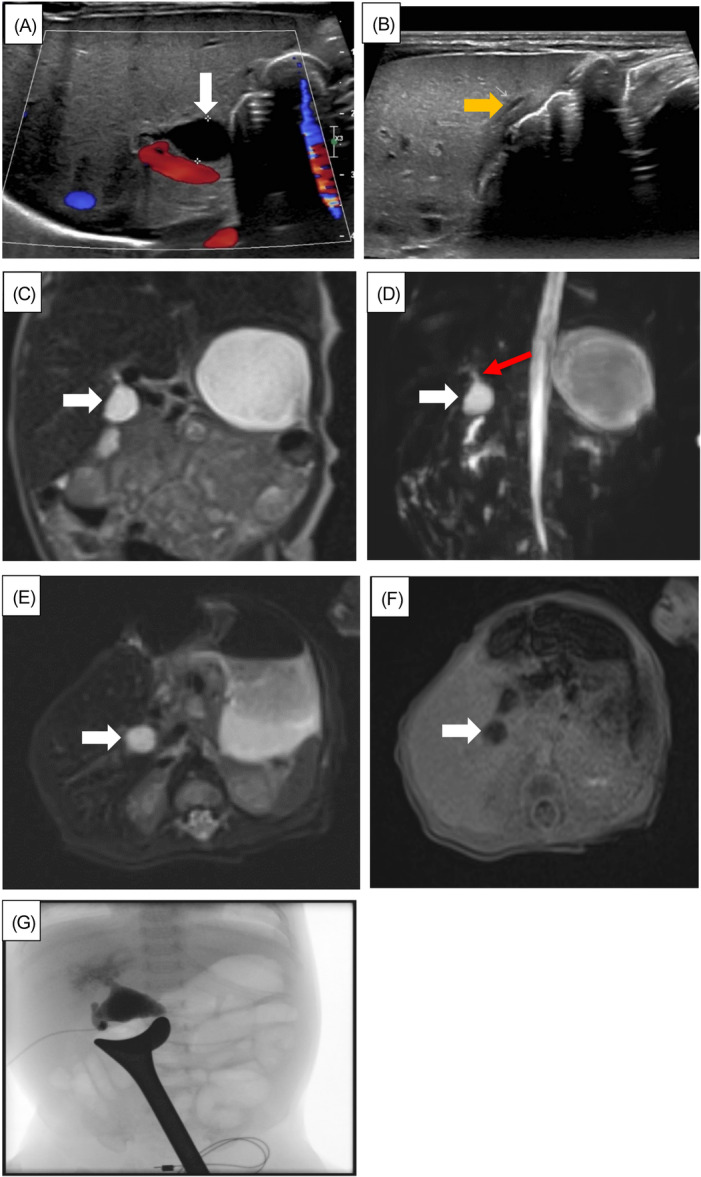
(A) Ultrasound Doppler of the liver revealing cystic structure in the porta hepatitis in continuity with the nondilated intrahepatic biliary system (white arrow). (B) Ultrasound Doppler of the liver, revealing a small gallbladder (orange arrow) was also present, which can be seen in BA. (C,D) T2‐weighted image coronal image from MRCP (C) and 3D maximum intensity projection MRI (D) demonstrating the cystic structure in the portal hepatis, which appears to communicate with the common hepatic duct. (D) Red arrow annotates a small proximal bile duct measuring 1.3 mm. (E,F) Axial T2 fat‐saturated image (E) and axial T1 20 min postcontrast delay image (F) after administration of hepatobiliary contrast agent, Eovist. The cystic structure remains dark on T1 and fails to opacify with excreted contrast. (G) Intraoperative cholangiography revealing that the gallbladder communicates with the proximal cystic structure that communicates with the left and right hepatic ducts. No evidence of distal flow into the duodenum is noted, suggestive of cystic variant of BA. 3D, three‐dimensional; BA, biliary atresia; MRCP, magnetic resonance cholangiopancreatography; MRI, magnetic resonance imaging.

A 2275 g, large for gestational age, female was born at 32^+2^ weeks of gestational age to a 30‐year‐old female, Gravida 4 Parity 3, via cesarean section secondary to maternal respiratory failure due to COVID‐19 pneumonia. The infant required continuous positive airway pressure at the delivery. APGAR (Appearance, pulse, grimace, activity, respiration) score was 7 and 9 at 1 and 5 min, respectively. The infant was admitted to the neonatal intensive care unit for management of respiratory distress and prematurity. Due to ABO incompatibility (maternal blood Group O positive; infant's blood Group B positive), total and direct (T&D) bilirubin were measured at 12 h of life, which were 5.1 and 0.9 mg/dL, respectively. The infant received a total of 12 h of phototherapy. Over the next few days, the infant's T&D bilirubin levels trended upwards. On Day of Life (DOL) 3, the infant's T&D bilirubin levels were 7.1 and 1.3 mg/dL, respectively. Ultrasound (US) of the abdomen was obtained, which was suggestive of Type 1 CC (Figure 1A,B); prenatal ultrasonographic information was not available for comparison purposes. Her gamma‐glutamyl transferase (GGT) level on DOL 4 was 1044 U/L. On DOL 6, magnetic resonance cholangiopancreatography (MRCP) with Eovist was obtained, which revealed a 1.2 cm cystic structure in the porta hepatis connecting to an abnormal slit‐like gallbladder (Figure 1C,D), and no excretion of Eovist into the biliary system (Figure 1E,F). A small proximal bile duct (PBD) was present, measuring 1.3 mm, but a distal common bile duct was not visualized (Figure 1D). This constellation of findings was more concerning for CBA over CC. A confirmatory cholangiogram was recommended. The infant was started on ursodiol and fat‐soluble vitamin supplements. On DOL 23, US‐guided liver biopsy revealed cholestasis and acute focal cholangitis with bile duct plugging concerning for early BA. However, these findings are not pathognomonic for BA and can also be seen in CC; hence, on DOL 46, the infant underwent intraoperative cholangiogram, which confirmed the diagnosis of Type III cystic BA (Figure 1G) and subsequently had KHPE with excision of extrahepatic cystic BA and Roux‐en‐Y hepaticojejunostomy. The pathology of the excised specimen revealed chronic cystitis with denuded mucosal surface. Due to the history of prematurity and low birth weight, the liver biopsy and intraoperative cholangiogram were delayed in this case. Her postoperative clinical course was uneventful, and she was discharged home on DOL 55. She currently follows our transplant hepatology team. She is currently 2 years old, growing appropriately, and has age‐appropriate developmental milestones. She has normal native liver synthetic function without clinically significant portal hypertension.

### 2.3 Case 2

**Figure 2:**
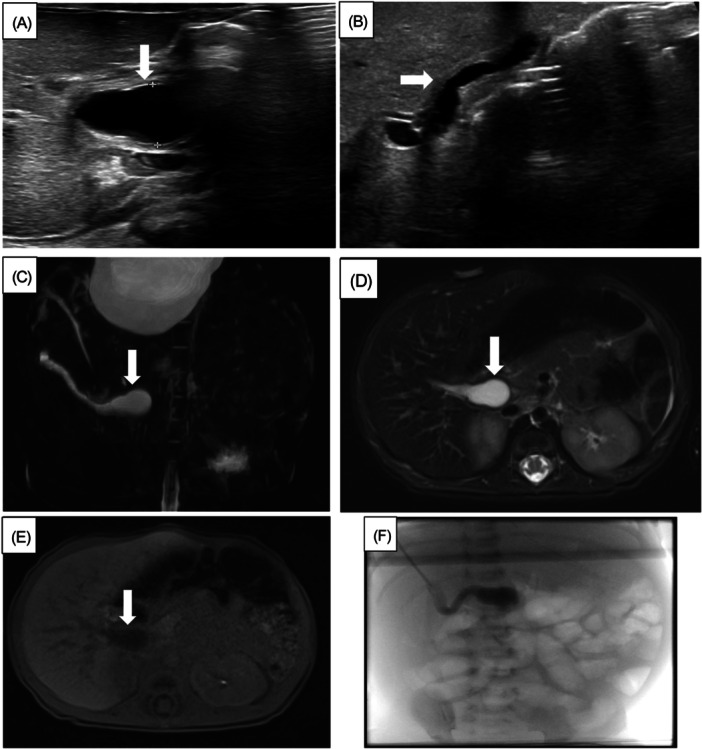
(A, B) Ultrasound Doppler of the liver revealing no dilated intrahepatic biliary ducts with fusiform avascular enlargement of the common bile duct suggestive of Type 1 choledochal cyst (A), and the gallbladder is normal in length measuring 2.5 cm (B). (C) 3D maximum intensity projection image from MRCP demonstrating a fusiform cystic structure in the porta hepatis communicating with the tubular gallbladder. (D, E) Axial T2 fat‐saturated image (D) and axial T1 20 min postcontrast delay image (E) after administration of Eovist. The cystic structure remains dark on T1 and fails to opacify with excreted contrast. (F) Cholangiography revealing patent gallbladder and infrahepatic cyst in the location of the common bile duct with atretic segments at the ampulla as well as the hepatic duct bifurcation, with no bile in the cystic structure, suggestive of cystic variant of BA. 3D, three‐dimensional; BA, biliary atresia; MRCP, magnetic resonance cholangiopancreatography.

A 17‐day‐old infant presented for further evaluation of direct hyperbilirubinemia. She was born at 39^+2^ weeks of gestational age via spontaneous vaginal delivery after induction of labor due to polyhydramnios. Prenatal anatomy scan at 13 weeks of gestational age was concerning for pancreatic and choroid plexus cysts; the choroid plexus cyst resolved on repeat scan. Postnatally, the infant required phototherapy for 3 days secondary to hyperbilirubinemia. The infant initially had clay colored stool, which later transitioned to pigmented stools. On DOL 10, the infant's T&D bilirubin were 18.4 and 2.4 mg/dL, respectively, with a GGT of 1251 U/L. She was started on ursodiol and fat‐soluble vitamin supplements, and abdominal US was obtained, which was suggestive of Type 1 CC with fusiform enlargement of the common bile duct (Figure 2A,B). Hence, MRCP with Eovist was performed on DOL 17, which revealed a 2.3 cm cystic structure in the porta hepatis connecting to a small caliber gallbladder (Figure 2C), and no excretion of Eovist into the biliary system was noted (Figure 2D,E). No common bile duct was visualized proximally nor distally to the cystic structure. This constellation of findings was concerning for CBA. However, given the relatively large size of the cyst, the interpreting radiologist felt CC could not be fully excluded, and a cholangiogram was recommended for confirmation. On DOL 20, infant underwent intraoperative cholangiogram, which confirmed the diagnosis of Type III cystic BA (Figure 2F) and subsequently underwent KHPE with excision of cystic BA and Roux‐en‐Y hepaticojejunostomy. Wedge liver biopsy obtained during the procedure revealed cholestatic hepatitis with ductular reaction consistent with BA. The gallbladder was partially denuded, and the biliary cyst was completely denuded with reactive changes. The postoperative clinical course was uneventful, the infant was discharged home on DOL 26, and direct bilirubin normalized at 4 months of age. Post KHPE course was complicated by compensated advanced chronic liver disease and with normal liver synthetic function and portal hypertension with caput medusae. As part of liver transplantation evaluation, liver biopsy was performed at 6 months of age, which was significant for bridging fibrosis and nodular regeneration consistent with liver cirrhosis. She ultimately underwent living living‐related donor liver transplant at 11 months of age. Her post‐transplant course has been complicated by Epstein–Barr virus and cytomegalovirus viremia, thrombocytopenia, portal vein stenosis, and gastrointestinal bleeding. She is currently 2 years old, growing appropriately with normal liver synthetic function, and hemodynamically insignificant mild portal vein stenosis.

## 3 DISCUSSION

There are different categorizations of BA; the congenital form accounts for 20% of BA and has syndromic associations, whereas the acquired form is more common and is the result of interaction between the innate and adaptive immune responses and viral triggers.^2^, ^5^ However, from a clinical perspective, the etiological heterogeneity of BA can be categorized into isolated type (the most common type accounting for 70%–80% of cases of BA), syndromic BA (consisting of BA with splenic malformation syndrome, cat‐eye syndrome, and “nonsyndromic” associated with, e.g., esophageal atresia, jejunal atresia, cleft palate etc.) cystic BA, and cytomegalovirus‐associated BA.^6^

During the fourth week of gestation, bile ducts initially develop as an outgrowth of the ventral foregut.^7^ Later in the ninth week, it realigns at the porta hepatitis to form connections with the liver and starts supporting the drainage of bile flow during 12th–13th week of gestation.^7^ Hence, one theory is that congenital BA starts developing in the early first trimester.^7^ There are three morphological subtypes of BA based upon biliary obliteration at the most proximal level: in Type 1, biliary obliteration starts at the level of the proximal cystic duct and common bile duct; in Type 2, biliary obliteration starts at the level of the common hepatic duct; and in Type 3, the extrahepatic biliary obliteration starts at the porta hepatis.^8^

BA is the most common cause of obstructive cholestasis, the exact etiology and pathogenesis of which is unknown.^2^ The most common presenting features of BA include conjugated hyperbilirubinemia, dark urine, acholic stool, hepatosplenomegaly, and liver cirrhosis.^9^ Early conjugated hyperbilirubinemia is emerging as an important diagnostic marker for BA with high sensitivity and specificity.^10^ Our patient in Case 2 had pigmented stool upon presentation, which made the clinical diagnosis of obstructive cholestasis difficult, but given elevated conjugated hyperbilirubinemia, we proceeded with the confirmatory investigations.

KHPE is considered to be the first‐line treatment for BA.^11^ Five years post KHPE native liver survival rate (NLSR) is around 40% due to progressive liver injury.^12^ KHPE should ideally be performed within DOL 30–45 for optimal success.^3^ Both of our infants underwent KHPE; the infant in Case 1 was unable to have KHPE in the recommended period of DOL 30–45 due to prematurity.

Early diagnosis of BA is critical for performing timely KHPE procedure, which can be made difficult by the existence of multiple variants of BA. CBA is one such variant that has a higher NLSR but is difficult to diagnose because of clinical and sonographic resemblance to CC.^4^, ^13^ The typical sonographic findings of BA include absent extrahepatic bile ducts, dysmorphic gallbladder, a positive triangular cord sign, increased hepatic artery diameter, and increased hepatic subcapsular flow.^14^ The presence of a microcyst in the porta hepatis is also specific for BA.^15^ However, a larger size cyst extending into the extrahepatic space makes it difficult to distinguish CBA from CC, as was the case in the presented patients.^15^, ^16^ The sonographic studies in our cases were felt to be inconclusive. The prenatal anatomy scan for Case 2 was suggestive of a pancreatic cyst, while the postnatal ultrasonography in both of our cases was initially felt to be more suggestive of CC rather than CBA, which made the diagnosis difficult. In such situations, hepatobiliary scintigraphy and magnetic resonance imaging (MRI) can aid in the preoperative diagnosis; however, hepatobiliary scintigraphy has only moderate specificity, and MRI has high sensitivity but low specificity.^14^ In our cases, the MRCP was helpful to suggest CBA, but out of an abundance of caution and technical limitations of MRCP in small babies, conventional cholangiograms were still advised for definitive diagnosis.

Use of a hepatobiliary‐specific contrast agent, such as Eovist (gadoxetate disodium), can potentially aid in the visualization of the biliary tree.^17^ The agent is actively transported into functioning hepatocytes. Eovist is accumulated in cells with normal hepatobiliary function and is not accumulated in lesions with little or no hepatobiliary function, which is valuable in the characterization of hepatic lesions and evaluation for hepatic metastases.^17^ Eovist is also excreted by hepatocytes into the biliary system on the 20 min delayed phase, which can aid in the diagnosis of CCs and biliary leaks.^17^ In the presented cases, if contrast had been excreted into the biliary tree and cystic structure and had drained into the duodenum, then CBA would have been excluded, and CC confirmed. In BA, Eovist fails to be excreted into the biliary system nor drain into the duodenum, mimicking the findings expected on hepatobiliary iminodiacetic acid (HIDA) scan. Unfortunately, one caveat to the use of Eovist is potentially poor uptake and excretion of Eovist in the setting of elevated serum bilirubin (>3 mg/dL), hence limiting the diagnostic quality of the exam.^18^ The two presented cases demonstrated no Eovist excretion, which, in conjunction with other findings, served as additional evidence favoring CBA. However, serum total bilirubin levels of 18.4 and 7.1 mg/dL, respectively, rendered the absence of Eovist excretion a less reliable finding, and CC could not be definitely excluded based solely on the absence of opacification. Therefore, CC can be ruled in when excreted Eovist opacifies the cyst and drains into the duodenum, but the absence of opacification should be interpreted with caution in the setting of elevated bilirubin. Therefore, more definitive diagnosis with percutaneous or intraoperative cholangiography is still needed in some cases.

He et al. further investigated MRCP findings that may aid in the differentiation of CBA and CC.^19^ Presence of abnormal connections between intrahepatic biliary ducts, called loop visualization, on three‐dimensional (3D) reconstructions was only seen in cases of CBA and not demonstrated in CC.^19^ Unfortunately, neither of the presented cases had 3D reconstructions available for review, and Case 1 was also premature, further limiting spatial resolution. Nonetheless, PBD diameter ≤1.3 mm was found to favor CBA over CC. The PBD measured 1.3 mm in Case 1 and 0 mm in Case 2. Cyst wall thickness >1 mm also favored CBA over CC, and measured 1.3 and 1.0 mm in the presented cases, respectively. Alternatively, the presence of a larger porta hepatis cyst (median 4.1 cm) and the presence of sludge favored CC. Percutaneous or intraoperative cholangiography remains the gold standard to confirm the diagnosis of CBA.^20^ Both of our CBA cases were confirmed via intraoperative cholangiogram. Caponcelli et al. reported in their study the absence of epithelial lining in the cyst of all their CBA cases, which was a consistent finding in both of our cases.^4^ Both of our cases presented early; hence, it was reasonable to obtain MRCP, which is a less invasive study, before proceeding to cholangiography. However, if the clinical concern is high and the infant presents late with concern for losing the optimal window to perform KHPE, cholangiography can be performed without obtaining MRCP first.

Lastly, there is literature regarding the use of endoscopic retrograde cholangiopancreatography (ERCP) to diagnose BA in infants with prolonged cholestasis, especially when noninvasive imaging is inconclusive. ERCP is a minimally invasive procedure that allows direct visualization of the biliary tree, which can help differentiate BA from other causes of neonatal cholestasis. Petersen et al. reported that ERCP had a sensitivity of 92% and specificity of 73% for diagnosing BA, and it avoided unnecessary surgical procedures in approximately 25% of cases.^21^ Similarly, Shteyer et al. found that ERCP led to the diagnosis of BA in 13 out of 27 infants, preventing more invasive procedures in some cases.^22^ Keil et al. also highlighted the high diagnostic accuracy of ERCP, with a sensitivity of 86% and specificity of 94% for BA.^23^

## 4 CONCLUSION

Our manuscript stresses upon how CBA can resemble CC and the importance of timely differentiation and diagnosis of the two bile tract entities, as the surgical interventions are different for them. In addition, our manuscript also highlights the importance of early diagnosis of CBA, given the optimum time window for performing KPHE for successfully salvaging the native liver.

## CONFLICT OF INTEREST STATEMENT

The authors declare no conflicts of interest.
